# Systematic review reveals lack of quality in reporting health-related quality of life in patients with gastroenteropancreatic neuroendocrine tumours

**DOI:** 10.1186/s12955-016-0527-2

**Published:** 2016-09-10

**Authors:** Caroline Martini, Eva-Maria Gamper, Lisa Wintner, Bernhard Nilica, Barbara Sperner-Unterweger, Bernhard Holzner, Irene Virgolini

**Affiliations:** 1 Department for Psychiatry, Psychotherapy and Psychosomatics, Medical University of Innsbruck, Anichstraße 35, 6020 Innsbruck, Austria; 2 Department for Nuclear Medicine, Medical University of Innsbruck, Anichstraße 35, 6020 Innsbruck, Austria

**Keywords:** Health-related quality of life, Gastroenteropancreatic neuroendocrine tumours, Patient-reported outcomes, Systematic review, Methodological quality

## Abstract

**Background:**

Gastroenteropancreatic neuroendocrine tumours (GEP-NET) are often slow-growing and patients may live for years with metastasised disease. Hence, along with increasing overall and progression-free survival, treatments aim at preserving patients’ well-being and health-related quality of life (HRQoL). However, studies on systematic HRQoL assessment in patients with GEP-NET are scarce. Therefore, the purpose of the current review is to systematically evaluate the methodological quality of the identified studies.

**Methods:**

A targeted database search was performed in PubMed, EMBASE, and CENTRAL. Data extraction was conducted by two independent researchers according to predefined criteria. For study evaluation, the Minimum Standard Checklist for Evaluating HRQoL Outcomes in Cancer Clinical Trials and the CONSORT Patient-Reported Outcome extension were adapted.

**Results:**

The database search yielded 48 eligible studies. We found the awareness for the need of HRQoL measurement to be growing and application of cancer-specific instruments gaining acceptance. Overall, studies were too heterogeneous in terms of patient characteristics and treatment interventions to draw clear conclusions for clinical practice. More importantly, a range of methodological shortcomings has been identified which were mainly related to the assessment and statistical analysis, as well as the reporting and interpretation of HRQoL data.

**Conclusion:**

Despite an increasing interest in HRQoL in GEP-NET patients, there is still a lack of knowledge on this issue. A transfer of HRQoL results into clinical practice is hindered not only by the scarceness of studies, but also by the often limited quality of HRQoL processing and reporting.

**Electronic supplementary material:**

The online version of this article (doi:10.1186/s12955-016-0527-2) contains supplementary material, which is available to authorized users.

## Background

With an incidence of 5.25 per 100,000 [1] gastroenteropancreatic neuroendocrine tumours (GEP-NET) are a relatively rare disease. They are usually slow-growing and often do not produce clear symptoms until they are metastasised. Currently, surgical tumour resection is the only curative treatment, and usually only in patients with localised disease [1–4]. The primary aims of available therapeutic options are to provide symptom relief, control tumour growth, improve long-term survival, and, not least, preserve psychosocial well-being and health-related quality of life (HRQoL) [2, 4].

The past two decades have shown that the patient’s subjective perspective on his/her own health, quality of life, and treatment-related aspects plays an essential role in treatment evaluation. Traditional physician proxy ratings on morbidity correlate only poorly with several self-reported functional capacity and well-being domains [5] – areas which are of utmost importance for the individual patient in managing his/her everyday life with the disease. These aspects are represented in the concept of HRQoL. There is agreement that HRQoL describes “the extent to which one’s usual or expected physical, emotional, and social well-being is affected by a medical condition or its treatment” ([6], p. 73) and needs to be assessed as a patient-reported outcome (PRO). PROs comprise any self-report of a patient’s health status without interpretation by a third person [7].

The assessment of HRQoL as an important secondary outcome in clinical studies, using reliable and valid self-report instruments, has become the criterion standard in oncology [8–10]. To date, systematic assessments of HRQoL in patients with GEP-NET have hardly been performed. High-quality information on HRQoL serves a variety of purposes, from the development of targeted interventions to informed decision making about treatment options to the allocation of healthcare resources [11].

Based on the assumptions above, we conducted a systematic review on studies incorporating HRQoL in patients with GEP-NET to evaluate the methodological quality of HRQoL processing and reporting. In detail, this review aims at investigating (i) the amount of available information on HRQoL in patients with GEP-NET, (ii) how HRQoL was assessed and reported, and (iii) if the quality of HRQoL information provided meets agreed standards.

## Methods

We applied a systematic approach to identify and appraise studies on HRQoL in GEP-NET patients. Criteria for the selection, description and evaluation of studies were based on the PRISMA (Preferred Reporting Items of Systematic Reviews and Meta-Analyses) checklist [12], the Cochrane Consumers and Communication Review Group’s Extraction Template [13], and the Minimum Standard Checklist for Evaluating HRQoL Outcomes in Cancer Clinical Trials [14].

### Search strategy

A systematic literature search was conducted in September 2014 and updated in July 2016 using PubMed, EMBASE, and CENTRAL. We searched for original research articles published in English, Italian, French, or German and restricted the search to studies on humans. Combinations of the following MeSH and free text terms were used: neuroendocrine tumors, digestive system neoplasms, neuroendocrine, endocrine, NET, foregut, midgut, hindgut, quality of life, patient-reported, self-reported, well-being, psycho*. An exemplary search history for PubMed is provided in Appendix.

In addition, we also searched OpenGrey (http://www.opengrey.eu) and BIOSIS previews (http://www.dimdi.de/static/de/db/dbinfo/ba70.htm) for the identification of grey literature. Database searches were augmented by a manual search of reference lists of included articles to identify further eligible studies not detected by our search terms.

### Inclusion criteria and data extraction

Due to the scarceness of studies on the topic of HRQoL in patients with GEP-NET, it was decided not to impose restriction criteria concerning study design in order to comprehensively capture the available information. Any study with a quantitative approach assessing self-reported HRQoL was considered eligible. Publications that were letters, editorials, narrative reviews, and case reports were excluded. Methodological studies on HRQoL in this patient group (e.g., instrument development) were considered not eligible. A data collection form was based on the Cochrane Consumers and Communication Review Group’s Extraction Template, adapted to study requirements, pilot tested on five randomly selected reports and refined accordingly.

Eligibility assessment and data extraction were performed by two independent researchers. In the case of disagreement, a third reviewer was consulted to reach final consensus. For the assessment of interrater agreement a simple Kappa calculation was performed, with values between .61 and .80 indicating substantial and of > .81 almost perfect agreement [15].

### Study evaluation

For study evaluation we adapted the Minimum Standard Checklist for Evaluating HRQoL Outcomes in Cancer Clinical Trials [14] and the Consolidated Standards of Reporting Trials (CONSORT) PRO extension checklist [16] for our requirements (i.e., applicable to a range of different study designs in addition to randomised controlled trials (RCTs)). Multiple reports on the same study were linked together and were subsequently defined as one study.

### Quality criteria were defined according to study design

For all studies it was documented if:

- HRQoL was identified as primary or secondary outcome in the abstract (for studies with more than one outcome parameter),
- there was an a priori hypothesis concerning HRQoL (not applicable if explorative),
- an explicit rationale for HRQoL instrument selection was provided,
- the instrument was validated or psychometric properties were reported or referenced,
- the HRQoL instrument was cancer-specific,
- the instrument administration was reported,
- missing data was documented or discussed,
- statistical considerations for dealing with missing data were provided,
- reports on HRQoL results were complete and scoring was correct,
- the issue of clinical significance had been addressed.

For cross-sectional studies it was additionally documented if:

- compliance was reported.

For all kinds of prospective studies it was additionally documented if:

- baseline compliance was documented,
- the timing of assessment was reported.

For prospective and comparative studies it was additionally documented if:

- statistical power or effect sizes for HRQoL results were reported.

For studies with HRQoL as secondary outcome it was additionally documented if:

- HRQoL results were considered in discussion section.

## Results

### Study selection and study characteristics

**Fig. 1:**
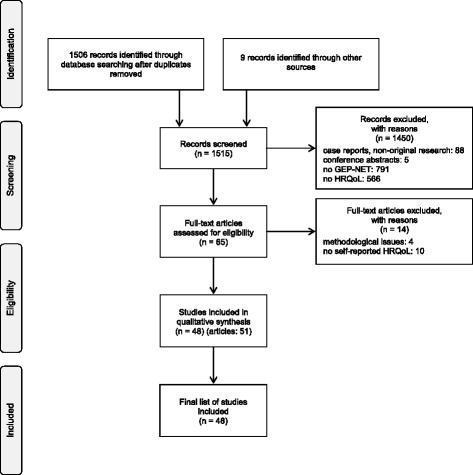
Flow-diagram of study selection process

The literature search yielded a total of 1506 records (after removing duplicates). Figure 1 shows the flow-diagram of the selection process. We screened titles and abstracts and excluded 88 case-reports and non-original research reports such as comments and letters. Subsequently, 791 studies were excluded for not investigating GEP-NET, and 566 were excluded for not investigating HRQoL. Grey literature search yielded five conference abstracts reporting on studies dealing with HRQoL issues which were not (or not yet) published as full reports and, therefore, not included in the review. Four additional studies were identified by hand search of reference lists of relevant articles. After these selection steps, 65 potentially relevant full-text articles remained and were assessed for eligibility. Out of these, 14 articles were excluded from the review for the following reasons: ten did not measure self-reported HRQoL (e.g., used the Karnofsky Performance Score), and four were methodological studies either on HRQoL instrument development or comparison of instruments. With a Kappa of .818 (*p* ≤ .001) the level of agreement between the two reviewers concerning inclusion or exclusion of a full-text article assessed for eligibility was high.

In total, 51 articles, which reported on 48 separate studies, were included. Eight were RCTs which assessed HRQoL as a secondary outcome to the primary endpoints response, progression-free survival, or time to progression. Additionally, 25 prospective studies, including 15 phase II trials, and 15 observational cross-sectional studies were identified.

Sample sizes ranged between 9 and 663 patients with a median of 51. Most of the studies investigated patients in an advanced stage of disease and administered the European Organisation for Research and Treatment of Cancer Quality of Life Questionnaire Core 30 (EORTC QLQ-C30) for assessing HRQoL. In total, 22 studies used either HRQoL or another PRO (e.g., fear of recurrence) as a primary outcome measure. Eight studies compared HRQoL scores with normative values from the national general population. For details on study characteristics see Table 1.

**Table 1:** Study characteristics of identified studies

Author, year	Sample size, diagnosis (disease severity)	Treatment modality/intervention	Comparison	PRO primary outcome	HRQoL measure
Randomised controlled trials					
Arnold et al., 2005 [52]	*N* = 109, foregut, midgut NET, CUP (locally advanced, metastatic)	octreotide	octreotide + INT	no	QLQ-C30
Bajetta et al., 2006 [53]	*N* = 60, well-differentiated intestinal, pancreatic, bronchial NET, other, CUP (low-grade malignancy)	lan ATG	lan MP	no	QLQ-C30
Caplin et al., 2014 [42] *(CLARINET)*	*N* = 204, well- or moderately differentiated pancreatic, midgut, hindgut NET, other, CUP (progressive, metastatic)	lan ATG	placebo	no	QLQ-C30, QLQ-GI.NET21
Jacobsen & Hanssen, 1995 [54]	*N* = 11, intestinal NET, PNET (liver metastases)	octreotide	placebo (cross-over design)	no	GHQ-30, PAIS
Meyer et al., 2014 [55]	*N* = 86, PNET, GI foregut NET, CUP (advanced, metastatic)	capecitabine + streptozocin + cisplatine	capecitabine + streptozocin	no	QLQ-C30
Raymond et al., 2011 [56]	*N* = 171, well-differentiated PNET (advanced, metastatic)	sunitinib	placebo	no	QLQ-C30
Rinke et al., 2009 [57] *(PROMID)*	*N* = 85, well-differentiated midgut NET (metastatic)	octreotide LAR	placebo	no	QLQ-C30
Yao et al., 2016 [45] *(RADIANT-4)*	*N* = 302, well-differentiated GI-NET, bronchopulmonary NET (advanced, progressive)	everolimus	placebo	no	FACT-G
Phase II studies					
Bodei et al., 2011 [58]	*N* = 51, bronchial, pancreatic, duodenal, ileal, appendicular, sigma-rectal NET, CUP (progressive, other)	^177^Lu-DOTATATE	-	no	QLQ-C30
Bushnell et al., 2010 [59]	*N* = 90, carcinoid (metastatic, refractory to octreotide)	^90^Y-DOTADOC	-	no	EQ-5D
Claringbold et al., 2011 [60]	*N* = 33, well-differentiated NET (progressive)	^177^Lu-DOTATATE	-	no	QLQ-C30
Cwikla et al., 2010 [61]	*N* = 60, GEP-NET (progressive, metastatic)	^90^Y-DOTATATE	-	no	QLQ-C30, QLQ-GI.NET21
Delpassand et al., 2014 [62]	*N* = 37, GEP-NET (progressive)	^177^Lu-DOTATATE	-	no	QLQ-C30
Ducreux et al., 2014 [43]; Mitry et al., 2014 [44] *(BETTER)*	*N* = 34, well-differentiated PNET (progressive, metastatic) *N* = 49, well-differentiated GI-NET (progressive, metastatic)	bevacizumab + 5-FU/streptozocin; bevacizumab + capecitabine	-	no	QLQ-C30
Frilling et al., 2006 [63]	*N* = 18, ileal, pancreatic NET, paraganglioma, gastrinoma, CUP (progressive, metastatic)	^90^Y-DOTATOC; ^177^Lu-DOTATOC	-	no	SF-36
Khan et al., 2011 [64]	*N* = 256, carcinoid, PNET, CUP, gastrinoma, glucagonoma, insulinoma, VIPoma (with and without metastases)	^177^Lu-DOTATATE	-	yes	QLQ-C30
Korse et al., 2009 [65]	*N* = 39, GI-NET (advanced, metastatic)	octreotide LAR	-	no	QLQ-C30
Kulke et al., 2008 [66]	*N* = 107, carcinoid, PNET (advanced)	sunitinib	-	no	EQ-5D, FACIT-Fatigue scale
Kvols et al., 2012 [67]	*N* = 45, GI-NET (advanced, metastatic)	pasireotide	-	no	FACIT-D
Martin-Richard et al., 2013 [68]	*N* = 30, well-differentiated GEP-NET, bronchopulmonary NET, CUP (progressive)	lan ATG	-	no	QLQ-C30
Ruszniewski et al., 2004 [69]	*N* = 71, foregut, midgut, hindgut NET, other (not terminally ill)	lan PR	-	no	QLQ-C30
Wymenga et al., 1999 [70]	*N* = 55, carcinoid, gastrinoma, VIPoma (tumour stages III and IV)	lan PR	-	no	QLQ-C30
Zuetenhorst et al., 2004 [71]	*N* = 26, well-differentiated ileo-cecal, gastric, bronchopulmonary NET, CUP (metastatic)	INT followed by unlabelled MIBG followed by ^131^I-MIBG	-	no	QLQ-C30
Prospective studies					
Fröjd et al., 2007 [72]; Fröjd et al., 2009 [73]	*N* = 36, carcinoid (metastatic, other)	INT; octreotide; INT + octreotide; CTX; octreotide + CTX; no treatment	normative data (subsample)	yes	QLQ-C30
Haugland et al., 2013 [74]	*N* = 37, GI-NET (not terminally ill)	medical treatment (n.s.)	-	yes	SF-36
Kalinowski et al., 2009 [75]	*N* = 9, bronchial, jejunal, ileal, gastric NET, PNET, insulinoma (liver metastases)	^90^Y microspheres	-	no	QLQ-C30, QLQ-LMC21
Kwekkeboom et al., 2003 [76]	*N* = 35, GEP-NET (progressive, other)	^177^Lu-DOTATATE	-	no	QLQ-C30
Larsson & Janson, 2008 [77]	*N* = 18, midgut carcinoid (n.s.)	INT	-	yes	QLQ-C30, FACT-An
Larsson et al., 2001 [78]	*N* = 24, midgut carcinoid (metastatic, other)	INT; SSA; INT + SSA	normative data	yes	QLQ-C30
O’Toole et al., 2000 [79]	*N* = 33, intestinal NET, PNET, other (metastatic)	octreotide followed by lanreotide	lanreotide followed by octreotide	no	ISPN
Pasieka et al., 2004 [80]	*N* = 24, small bowel carcinoid, medullary thyroid cancer, CUP (progressive, metastatic)	^131^I-MIBG; ^111^In-octreotide	-	no	ad hoc questionnaire
Spolverato et al., 2015 [81]	*N* = 85, intestinal, pancreatic, bronchial NET, CUP (neuroendocrine liver metastasis)	surgical vs. nonsurgical treatment	-	yes	self-constructed questionnaire
Teunissen et al., 2004 [82]	*N* = 50, carcinoid, PNET, CUP, gastrinoma, insulinoma (metastatic)	^177^Lu-DOTATATE	-	yes	QLQ-C30
Cross-sectional studies					
Beaumont et al., 2012 [83];	*N* = 663, carcinoid, islet cell, “do not know or not sure which type” (local, regional, distant, currently not present)	surgery; surgery + SSA; other; no past/current treatment	normative data	yes	SF-36, PROMIS global health short form, PROMIS-29
Pearman et al., 2016 [84]			-		
Gelhorn et al., 2016 [85]	*N* = 11, midgut, hindgut NET (metastatic)	telotristat etiprate	normative data	yes	QLQ-C30, QLQ-GI.NET21
Haugland et al., 2009 [86]	*N* = 96, GI-NET (not terminally ill)	INT; SSA; INT + SSA; CTX; no treatment	normative data	yes	SF-36
Haugland et al., 2016 [87]	*N* = 196, GI-NET (not terminally ill)	medical treatment (n.s.)	normative data	yes	SF-36
Larsson et al., 1998 [88]	*N* = 17, carcinoid, PNET (not terminally ill) (+ staff, *N* = 17)	INT; SSA; INT + SSA	-	yes	QLQ-C30
Larsson, Sjöden et al., 1999 [89]	*N* = 119, carcinoid, PNET (n.s.)	INT + octreotid; INT; octreotide; CTX; XTR; omeprazol; no treatment	-	yes	QLQ-C30
Larsson, von Essen et al., 1999 [90]	*N* = 99, carcinoid, PNET (not terminally ill)	INT; SSA; INT + SSA	-	yes	QLQ-C30
Larsson et al., 2003 [91]	*N* = 19, carcinoid (n.s.) (+ staff, *N* = 19)	INT; SSA; INT + SSA	-	yes	semi-structured interviews
Larsson et al., 2007 [92]	*N* = 83, carcinoid, PNET (n.s.)	INT/octreotide; CTX; XTR; omeprazol; no treatment	-	yes	QLQ-C30
Petzel et al., 2012 [93]	*N* = 240, PNET, periampullary neoplasms (disease-free after surgery)	no current treatment	-	yes	FACT-Hep
Pezzilli et al., 2009 [94]	*N* = 44 PNET (disease free, advanced)	SSA; SSA + other; no treatment	normative data	yes	SF-12, GHQ-12
Pezzilli et al., 2010 [95]	*N* = 44 ileal NET (disease free, advanced)	SSA; INT; CTX; SSA + other; no treatment		yes	SF-12
Ruszniewski et al., 2016 [96] *(SYMNET)*	*N* = 273, small bowel carcinoid, appendicular, colonic, bronchopulmonary NET, CUP (92 % with metastases)	lan ATG	-	yes	QLQ-C30, QLQ-GI.NET
van der Horst-Schrivers et al., 2009 [97]	*N* = 43, midgut NET (metastatic)	SSA; INT; SSA + INT	normative data	yes	QLQ-C30
von Essen et al., 2002 [98]	*N* = 85, GI-NET (n.s.)	INT; SSA; INT + SSA	-	yes	QLQ-C30
Grey literature					
Garcia-Hernandez et al., 2012 [99]	*N* = 74, GI-NET (n.a.)	n.a.	-	yes	QLQ-C30, QLQ-GI.NET21
Gyökeres et al., 2010 [100]	*N* = 93, GI-NET (59 % with metastases)	SSA	-	yes	QLQ-C30, QLQ-GI.NET21
Marinova et al., 2016 [101]	*N* = 68, PNET (n.a.)	PRRT (n.s.)	-	yes	QLQ-C30
Pavel et al., 2013 [102]	*N* = 126, PNET (advanced)	everolimus	-	no	QLQ-C30, QLQ-GI.NET21
Strosberg et al., 2015 [103]	*N* = 230, midgut NET (advanced, progressive, metastatic)	^177^Lu-DOTATATE	octreotide LAR	no	QLQ-C30, QLQ-GI.NET21

Note. *CUP* cancer with unknown primary, *CTX* chemotherapy, *GEP-NET* gastroenteropancreatic NET, *GI-NET* gastrointestinal NET, *INT* interferon-α, *lan ATG* lanreotide autogel, *lan MP* lanreotide microparticles, *lan PR* lanreotide prolonged-release, *MIBG* meta-iodbenzylguanidin, *NET* neuroendocrine tumours, *n.a.* not available, *n.s.* not specified, *octreotide LAR* octreotide long-acting repeatable, *PNET* pancreatic NET, *XTR* radiotherapy, *SSA* somatostatin analogues

### Applied instruments for measuring health-related quality of life

The majority of the reviewed studies (31/48) used the EORTC QLQ-C30 [17], which is one of the most widely used HRQoL questionnaire in oncology in Europe. It has been shown to have good validity and reliability and consists of 30 items incorporating five functional scales (physical, role, emotional, cognitive, social), three symptom scales (fatigue, nausea/vomiting, pain), six single items (dyspnoea, insomnia, appetite loss, constipation, diarrhoea, financial difficulties) and one scale assessing global health status/QoL. Scores are linearly transformed to a 0–100 scale with higher scores representing a higher level of functioning and a higher level of symptomatology, respectively. As defined by Osoba et al. [18], mean changes in HRQoL scores over time of 5 to 10 points are considered as “small”, 10 to 20 points as “moderate”, and more than 20 points as “large” with regard to clinical relevance. The QLQ-C30 can be supplemented with disease- and treatment-specific modules. The NET-specific module, the QLQ-GI.NET21 [19], was used in four of the reviewed studies. It covers issues specific for GEP-NET and is applicable to patients suffering from endocrine or gastrointestinal symptoms. One study used the QLQ-LMC21, which is a module for patients with liver metastases from a colorectal tumour.

The same principle of adding disease- and treatment-specific modules to a generic questionnaire is applied by the Functional Assessment of Chronic Illness Therapy (FACIT) measures, which is one of the most frequently used HRQoL measurement systems in the US and Canada. The core questionnaire, the Functional Assessment of Cancer Therapy-General (FACT-G) [20], is composed of 27 items assessing physical, emotional, functional and social well-being. The FACT-G was used in five studies; applied modules were the FACT-Hepatobiliary, the FACT-Anemia, the FACIT-Diarrhea, and the FACIT-Fatigue.

One study used the Patient-Reported Outcome Measurement Information System 29-item Health Profile (PROMIS-29) which measures HRQoL in seven domains (depression, anxiety, physical function, pain interference, fatigue, sleep disturbance, ability to participate in social roles and activities) and was designed for patients with a wide range of chronic diseases [21]. In another study the Psychosocial Adjustment to Illness Scale (PAIS) was used. The PAIS was designed as a semi-structured clinical interview assessing a patient’s psychosocial adjustment to medical illness in terms of multiple domains (health care orientation, vocational environment, domestic environment, sexual relationships, extended family relationships, social environment, psychological distress) [22].

Generic instruments, namely the 36-Item Short Form Health Survey (SF-36), the 12-Item Short Form Health Survey (SF-12), the Euroqol-5 Dimension (EQ-5D), the General Health Questionnaire 30 (GHQ-30) and 12 (GHQ-12), and the French version of the Nottingham Health Profile (ISPN), were used in 10 studies. These instruments assess physical, emotional, functional and social aspects that are applicable across patient groups and diseases and are therefore less suitable for the identification of cancer site- or problem-specific concerns [23].

### Evaluation of methodological quality of HRQoL reporting

**Fig. 2:**
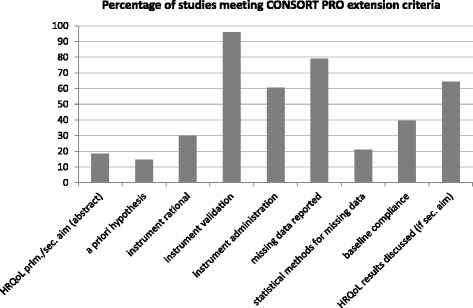
Percentage of studies meeting CONSORT PRO extension criteria

**Fig. 3:**
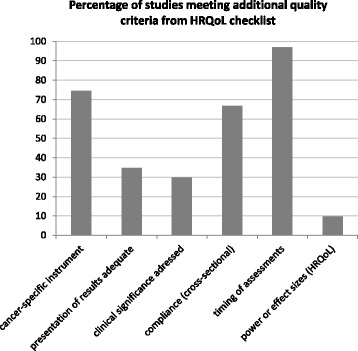
Percentage of studies meeting additional quality criteria from HRQoL checklist

Detailed information for each study based on the Minimum Standard Checklist for Evaluating HRQoL Outcomes in Cancer Clinical Trials and the CONSORT PRO extension checklist is provided in Table 2. Figure 2 shows the percentage of studies meeting the CONSORT PRO reporting criteria. The percentage of studies meeting additional quality criteria is depicted in Fig. 3. It should be noted that stated percentages do not always refer to all 48 extracted studies, but to the total number of studies that met the defined criteria.

**Table 2:** Study evaluation according to defined quality criteria

Author, year	HRQoL stated as prim./sec. aim^a^	A priori hypothesis	Instrument rational	Instrument validation^b^	Cancer-specific instrument	Instrument administration	(Baseline) compliance	Timing of assessments	Missing data reported	Statistical methods for missing data	Power or effect sizes (HRQoL)	Presentation of results adequate^c^	Clinical significance addressed	HRQoL results discussed
Randomised controlled trials														
Arnold et al., 2005 [52]	-	-	+	+	+	-	+	+	+	-	-	-	-	+
Bajetta et al., 2006 [53]	-	-	-	+	+	+	-	+	+	+	-	-	-	+
Caplin et al., 2014 [42] *(CLARINET)*	+	-	-	+	+	-	-	+	+	+	-	+	-	-
Jacobsen & Hanssen, 1995 [54]	+	-	-	+	-^d^	-	+	+	+	-	-	-	+	+
Meyer et al., 2014 [55]	+	-	-	+	+	-	+	+	+	-	-	-	-	-
Raymond et al., 2011 [56]	-	-	-	+	+	+	-	+	+	-	-	-	+	+
Rinke et al., 2009 [57] *(PROMID)*	-	-	-	+	+	-	+	+	-	-	-	-	-	-
Yao et al., 2016 [45] *(RADIANT-4)*	-	-	+	+	+	+	+	+	-	+	-	n.r.	+	n.r.
Phase II studies														
Bodei et al., 2011 [58]	-	-	-	+	+	-	-	+	-	-	-	-	-	+
Bushnell et al., 2010 [59]	-	-	-	+	-	-	+	+	+	-	-	-	-	-
Claringbold et al., 2011 [60]	-	-	-	+	+	-	-	+	+	-	-	-	-	+
Cwikla et al., 2010 [61]	-	-	-	+	+	+	-	+	+	-	-	-	-	-
Delpassand et al., 2014 [62]	-	-	-	+	+	-	-	+	+	-	-	-	-	-
Ducreux et al., 2014 [43]; Mitry et al., 2014 [44] *(BETTER)*	+	-	-	+	+	-	-	+	+	-	-	-	-	-
Frilling et al., 2006 [63]	-	-	-	+	-	-	-	+	+	-	-	-	-	-
Khan et al., 2011 [64]	N/A	-	+	+	+	+	+	+	+	-	-	-	+	+
Korse et al., 2009 [65]	N/A	-	+	+	+^d^	-	-	-	+	-	-	-	-	N/A
Kulke et al., 2008 [66]	-	-	-	+	-	+	-	+	+	-	-	-	-	+
Kvols et al., 2012 [67]	-	-	-	+	+	-	-	+	+	-	-	-	-	+
Martin-Richard et al., 2013 [68]	+	-	-	+	+	-	-	+	-	-	-	+	-	-
Ruszniewski et al., 2004 [69]	-	-	-	+	+	-	+	+	+	-	-	-	-	+
Wymenga et al., 1999 [70]	-	-	-	+	+	-	-	+	-	+	-	+	-	+
Zuetenhorst et al., 2004 [71]	-	+	-	+	+^d^	-	-	+	+	-	-	-	-	+
Prospective studies														
Fröjd et al., 2007 [72]; Fröjd et al., 2009 [73]	N/A	N/A	-	+	+^d^	+	+	+	+	+	-	+	+	N/A
Haugland et al., 2013 [74]	N/A	-	-	+	-	+	+	+	+	+	+	+	-	N/A
Kalinowski et al., 2009 [75]	-	-	+	+	+	-	-	+	-	-	-	-	-	+
Kwekkeboom et al., 2003 [76]	-	-	-	+	+	+	-	+	+	-	-	-	-	+
Larsson & Janson, 2008 [77]	N/A	N/A	+	+	+	-	-	+	+	-	-	+	+	N/A
Larsson et al., 2001 [78]	N/A	-	-	+	+^d^	+	+	+	+	-	-	+	+	N/A
O’Toole et al., 2000 [79]	-	+	-	+	-^d^	+	-	+	-	-	-	+	-	+
Pasieka et al., 2004 [80]	-	-	-	-	-	+	+	+	+	-	-	-	+	+
Spolverato et al., 2015 [81]	N/A	+	+	-	+	+	+	+	+	-	-	-	-	N/A
Teunissen et al., 2004 [82]	N/A	N/A	+	+	+	+	-	+	+	+	-	+	+	N/A
Cross-sectional studies														
Beaumont et al., 2012 [83];	N/A	N/A	-	+	-	+	+	N/A	+	-	+	+	+	N/A
Pearman et al., 2016 [84]													-	
Gelhorn et al., 2016 [85]	-	N/A	-	+	+	+	+	N/A	+	-	N/A	-	+	+
Haugland et al., 2009 [86]	N/A	N/A	+	+	-	+	+	N/A	+	-	+	+	+	N/A
Haugland et al., 2016 [87]	N/A	+	-	+	-	+	+	N/A	+	+	-	-	-	N/A
Larsson et al., 1998 [88]	N/A	N/A	+	+	+^d^	+	+	N/A	+	-	N/A	-	-	N/A
Larsson, Sjöden et al., 1999 [89]	N/A	N/A	+	+	+	+	-	N/A	+	-	N/A	+	+	N/A
Larsson, von Essen et al., 1999 [90]	N/A	N/A	+	+	+^d^	+	+	N/A	+	-	N/A	+	+	N/A
Larsson et al., 2003 [91]	N/A	N/A	N/A	N/A	N/A	+	-	N/A	+	N/A	N/A	N/A	N/A	N/A
Larsson et al., 2007 [92]	N/A	N/A	+	+	+^d^	+	+	N/A	+	-	N/A	+	-	N/A
Petzel et al., 2012 [93]	N/A	+	+	+	+	+	-	N/A	+	+	N/A	+	-	N/A
Pezzilli et al., 2009 [94]	N/A	-	-	+	-	+	+	N/A	+	-	-	-	-	N/A
Pezzilli et al., 2010 [95]	N/A	-	-	+	-	+	-	N/A	-	-	-	-	-	N/A
Ruszniewski et al., 2016 [96] *(SYMNET)*	-	N/A	-	+	+	+	-	N/A	-	+	+	-	-	+
van der Horst-Schrivers et al., 2009 [97]	N/A	N/A	-	+	+	+	+	N/A	-	-	-	+	-	N/A
von Essen et al., 2002 [98]	N/A	N/A	-	+	+	+	+	N/A	+	-	-	-	-	N/A

Note. ^a^in the abstract of the article; ^b^or psychometric properties reported; ^c^considered adequate if scoring has been performed correctly and if all assessed HRQoL domains were reported (including relevant *p*-values); ^d^additional ad hoc questions on symptoms added; *N/A* not applicable due to study design, *n.r.* not reported

Study evaluation revealed three major topics of concern. The first is a lack of knowledge on how to assess and process HRQoL data, and here particularly the absence of a priori hypotheses on HRQoL outcomes in 85 % and missing rationales for applied questionnaires in 70 % of studies. The second is a lack of adequate reporting of HRQoL results. For 65 % of studies, the presentation of results was rated inadequate, either due to incomplete reporting of HRQoL scores (only single scales or statistical significant results) or due to invalid score calculation (i.e., not according to the respective scoring manual). The third is related to the statistical methods as well as interpretation and discussion of HRQoL results. Information on the handling of missing data was not given in 79 % of studies. Less than one third of the studies (30 %) addressed the issue of clinical significance of findings. Further evaluation revealed that studies which investigated HRQoL as a primary outcome showed higher methodological quality of HRQoL data than those that included HRQoL as a secondary outcome measure. This was especially true for presentation and interpretation of results.

### Overview on HRQoL outcomes

From our literature search, we identified 8 RCTs investigating a broad range of patient groups regarding cancer site and stage, disease duration, as well as treatment modality. According to the predefined criteria, reporting of HRQoL data was of moderate to poor quality, especially in terms of completeness and adequate presentation of results. Thus, due to heterogeneity and methodological limitations of studies, no firm conclusions for clinical practice can be drawn.

There is evidence from non-RCTs including HRQoL as a primary outcome that patients with GEP-NET perceive their overall HRQoL as relatively good and stable. However, in-depth evaluation of these studies revealed a range of physical and psychosocial complaints primarily related to diarrhoea, flushing, and fatigue, as well as emotional, social, and role functioning when compared to the general population. Again, it has to be noted that these results should be interpreted with caution and not be considered as a basis for informing clinical practice and decision making. A brief summary of primary outcomes and HRQoL results of the reviewed studies is provided in Additional file 1.

## Discussion

The evaluation of HRQoL in patients with GEP-NET has attracted increasing interest in recent years. Despite this positive trend, however, there is still little knowledge available on this topic, and existing studies have major methodological limitations that hinder the application of HRQoL findings in daily clinical practice. The aim of this review was to evaluate the methodological quality of studies on HRQoL in GEP-NET patients, from data collection through to interpretation of results.

Our literature search yielded 58 abstracts ostensibly dealing with HRQoL issues in GEP-NET patients. However, further evaluation reveals that ten of these studies did not assess HRQoL at all but rather performed proxy ratings on physical functioning and self-care abilities. As the agreed definition of HRQoL is that it assesses a range of health issues beyond physical abilities and inevitably comprises patients’ self-reports [7, 24–26], these studies were excluded from the systematic review.

The evaluation of the remaining 48 studies, on a positive note, revealed a consistent application of appropriate HRQoL instruments. The majority of studies (35/48) used a cancer-specific questionnaire with already proven reliability and validity. This finding is in line with previous studies on the quality of PRO reporting in oncology RCTs, indicating that a majority of studies including PROs used validated disease- or at least cancer-specific instruments [27–29]. In the current review, nine studies amended these questionnaires with ad hoc questions on NET-specific symptoms, such as diarrhoea and hot flushes. It should be noted, however, that this was done in the absence of validated NET-specific PRO instruments. One study, however, administered an ad hoc instrument alone (i.e., not in combination with another validated measure) and another study used a self-constructed questionnaire comprising elements of validated measures. The term ‘ad hoc’ refers to the fact that these questions were not formally developed and had not undergone psychometric testing. In order to ensure high methodological quality and improve comparability of data across different studies, ad hoc questions should be considered only, if there are no appropriate validated PRO instruments [30, 31]. There is awareness of the fact that novel treatment agents may entail a range of symptoms not currently covered by validated PRO/HRQoL instruments [32–34] and methodological approaches of addressing this issue are being discussed. The EORTC Quality of Life Group (QLG) runs a database with an item pool including all questions from the disease-specific supplementary modules of the QLQ-C30, validated for cancer patients and different languages. Selecting suitable questions from such an item bank should be preferred over designing ad hoc questions. For patients with GEP-NET, two disease-specific questionnaires, the EORTC QLQ-GI.NET21 [19], a module to be applied together with the core questionnaire QLQ-C30, and the Norfolk QOL-NET [35], a NET-specific stand-alone measure, have become available only recently.

A range of methodological shortcomings were identified in assessing and processing as well as in reporting and interpreting HRQoL data. Most of the reviewed studies were heterogeneous in terms of study design and quality making it impossible to draw meaningful conclusions for clinical practice at this point. This refers primarily to studies investigating HRQoL as a secondary outcome. A large percentage of these studies provided only crude or incomplete presentations of HRQoL results or did not apply correct scoring procedures as prescribed by respective manuals. One of the included RCTs used HRQoL as a secondary outcome, as indicated in the clinical trial protocol as well as in the Methods section of the respective main publication, but failed to report on these issues both in the Results and Discussion section. Another common limitation was the lack of information on the statistical approach of handling missing HRQoL data, which was also found in previous reviews including different malignancies such as brain, bladder, prostate, and gynaecological cancers [27, 28, 36, 37]. These limitations were apparent even in high-evidence studies that are likely to impact on health policy and practice. None of the eight RCTs included in this review could provide HRQoL information in a way that would allow its use for informed decision making and planning future trials. Thus, while acknowledging the need for assessing HRQoL as an important secondary outcome in clinical studies, there seems to be an uncertainty about the processing of the collected data [14, 38–40]. Similar to the results of previous studies investigating the methodological quality of PRO reporting in patients with brain [27], prostate [36], and gynaecological [28] cancers, further problems were related to the interpretation of HRQoL results, especially regarding the clinical significance of HRQoL findings (e.g., changes over time, differences between treatment arms). Studies included in this review that used HRQoL as a primary outcome provided evidence that patients present with impairments in multiple domains such as emotional, role, and social functioning when compared to general population norms. This is in contrast to findings showing that GEP-NET patients generally perceived their HRQoL as relatively good. As mentioned above, to date, existing studies do not allow for firm conclusions and call for further research to elucidate the time course of HRQoL in this patient group.

Two recent review articles [29, 39] evaluating the methodological quality of PRO reporting in high-evidence studies on different cancer types indicated that the overall level of reporting according to the CONSORT PRO extension criteria was poor. Exceptions were studies assessing HRQoL as a primary outcome and/or presenting a supplementary report on HRQoL issues which showed a better performance. The overall quality of PRO reporting might benefit from increasing familiarity with HRQoL issues and the development of the above mentioned guidelines [29, 36, 39]. However, in spite of this development, a recent review on the consistency of available PRO-specific guidance has identified a clear lack of respective recommendations for the appropriate implementation of PROs in clinical research [41]. Although the checklists applied in this review [14, 16] do not represent an exhaustive set of criteria for high-quality HRQoL assessment for studies including HRQoL either as a primary or secondary outcome, their application both during study planning and reporting would substantially enhance the quality of the assessed data.

Therefore, while agreeing that further guidance is required to make HRQoL assessment more feasible and – with increasing quality – accessible for clinical use, we emphasise the need to adhere to already existing quality standards. Important scientific societies that have shaped the field of HRQoL research in oncology over the past two decades, such as the EORTC QLG, the FACIT group and the International Society for Quality of Life Research (ISOQOL), provide a well-informed basis for the application of PROs and should therefore be consulted when considering their incorporation into clinical trials.

In the field of GEP-NET research, there is a growing number of clinical trials considering HRQoL as an outcome measure [42–48]. However, two recent, not yet published, phase III trials fail to incorporate PRO or HRQoL assessments, as indicated in the respective study protocols [49, 50]. Furthermore, the methodological quality of information derived thus far is not satisfactory. Considering the fact that such studies have the potential to impact on health policy and practice, the importance of generating high-quality HRQoL data cannot be overstated. Poorly designed and/or reported PROs are likely to undermine the credibility of the results, which in turn hinders their application in daily clinical practice [9, 14, 27, 29, 37, 39, 40]. Especially with the movement towards a more patient-centred health care system, the incorporation of the patient’s subjective perspective plays a pivotal role in facilitating patient involvement in health care, thereby enhancing patient empowerment and satisfaction [51].

## Conclusions

Despite an increasing interest in assessing HRQoL in patients with GEP-NET, there is still little knowledge on the course of HRQoL over time, highlighting the need for high-quality longitudinal studies. Existing studies show methodological shortcomings in both processing and reporting of HRQoL data, especially when included as a secondary outcome in clinical trials. Methodological limitations were identified even for studies with high evidence level, which is considered problematic given their impact on health policy and medical practice. Thus, the valid application of HRQoL findings in clinical practice is hampered not only by lack of studies, but also by various methodological limitations of the existing ones. High-quality, well-reported HRQoL data is of utmost importance to make results accessible and useful to patients and their treating physicians. Therefore, it is strongly recommended to adhere to existing guidelines on the incorporation of PROs into clinical research.

## Appendix

**Table 3:** Exemplary search history for PubMed

Search	Add to builder	Query	Items found
#16	Add	Search (((((((neuroendocrine tumor[MeSH Terms]) OR ((digestive system neoplasms[MeSH Terms]) AND (((((neuroendocrine[Title/Abstract]) OR endocrine[Title/Abstract]) OR NET[Title/Abstract])) OR (((foregut[Title/Abstract]) OR midgut[Title/Abstract]) OR hindgut[Title/Abstract]))))) AND (((((quality of life) OR psycho*) OR well-being) OR patient-reported) OR self-reported))) NOT ((review) OR case-report))) NOT (((melanoma) OR schwannoma) OR ac*ustic neuroma) Filters: Classical Article; Clinical Study; Clinical Trial; Comparative Study; Controlled Clinical Trial; Multicenter Study; Observational Study; Randomized Controlled Trial; Humans; English; French; German; Italian; Spanish	796
#15	Add	Search (((((((neuroendocrine tumor[MeSH Terms]) OR ((digestive system neoplasms[MeSH Terms]) AND (((((neuroendocrine[Title/Abstract]) OR endocrine[Title/Abstract]) OR NET[Title/Abstract])) OR (((foregut[Title/Abstract]) OR midgut[Title/Abstract]) OR hindgut[Title/Abstract]))))) AND (((((quality of life) OR psycho*) OR well-being) OR patient-reported) OR self-reported))) NOT ((review) OR case-report))) NOT (((melanoma) OR schwannoma) OR ac*ustic neuroma) Filters: Classical Article; Clinical Study; Clinical Trial; Comparative Study; Controlled Clinical Trial; Multicenter Study; Observational Study; Randomized Controlled Trial; Humans	815
#14	Add	Search (((((((neuroendocrine tumor[MeSH Terms]) OR ((digestive system neoplasms[MeSH Terms]) AND (((((neuroendocrine[Title/Abstract]) OR endocrine[Title/Abstract]) OR NET[Title/Abstract])) OR (((foregut[Title/Abstract]) OR midgut[Title/Abstract]) OR hindgut[Title/Abstract]))))) AND (((((quality of life) OR psycho*) OR well-being) OR patient-reported) OR self-reported))) NOT ((review) OR case-report))) NOT (((melanoma) OR schwannoma) OR ac*ustic neuroma) Filters: Classical Article; Clinical Study; Clinical Trial; Comparative Study; Controlled Clinical Trial; Multicenter Study; Observational Study; Randomized Controlled Trial	858
#13	Add	Search (((((((neuroendocrine tumor[MeSH Terms]) OR ((digestive system neoplasms[MeSH Terms]) AND (((((neuroendocrine[Title/Abstract]) OR endocrine[Title/Abstract]) OR NET[Title/Abstract])) OR (((foregut[Title/Abstract]) OR midgut[Title/Abstract]) OR hindgut[Title/Abstract]))))) AND (((((quality of life) OR psycho*) OR well-being) OR patient-reported) OR self-reported))) NOT ((review) OR case-report))) NOT (((melanoma) OR schwannoma) OR ac*ustic neuroma)	4198
#12	Add	Search (((((neuroendocrine tumor[MeSH Terms]) OR ((digestive system neoplasms[MeSH Terms]) AND (((((neuroendocrine[Title/Abstract]) OR endocrine[Title/Abstract]) OR NET[Title/Abstract])) OR (((foregut[Title/Abstract]) OR midgut[Title/Abstract]) OR hindgut[Title/Abstract]))))) AND (((((quality of life) OR psycho*) OR well-being) OR patient-reported) OR self-reported))) NOT ((review) OR case-report)	15865
#11	Add	Search ((melanoma) OR schwannoma) OR ac*ustic neuroma	132755
#10	Add	Search (review) OR case-report	4299707
#9	Add	Search (((neuroendocrine tumor[MeSH Terms]) OR ((digestive system neoplasms[MeSH Terms]) AND (((((neuroendocrine[Title/Abstract]) OR endocrine[Title/Abstract]) OR NET[Title/Abstract])) OR (((foregut[Title/Abstract]) OR midgut[Title/Abstract]) OR hindgut[Title/Abstract]))))) AND (((((quality of life) OR psycho*) OR well-being) OR patient-reported) OR self-reported)	24382
#8	Add	Search (neuroendocrine tumor[MeSH Terms]) OR ((digestive system neoplasms[MeSH Terms]) AND (((((neuroendocrine[Title/Abstract]) OR endocrine[Title/Abstract]) OR NET[Title/Abstract])) OR (((foregut[Title/Abstract]) OR midgut[Title/Abstract]) OR hindgut[Title/Abstract])))	151289
#7	Add	Search (digestive system neoplasms[MeSH Terms]) AND (((((neuroendocrine[Title/Abstract]) OR endocrine[Title/Abstract]) OR NET[Title/Abstract])) OR (((foregut[Title/Abstract]) OR midgut[Title/Abstract]) OR hindgut[Title/Abstract]))	11944
#6	Add	Search ((((neuroendocrine[Title/Abstract]) OR endocrine[Title/Abstract]) OR NET[Title/Abstract])) OR (((foregut[Title/Abstract]) OR midgut[Title/Abstract]) OR hindgut[Title/Abstract])	241029
#5	Add	Search ((((quality of life) OR psycho*) OR well-being) OR patient-reported) OR self-reported	5592512
#4	Add	Search ((foregut[Title/Abstract]) OR midgut[Title/Abstract]) OR hindgut[Title/Abstract]	12719
#3	Add	Search ((neuroendocrine[Title/Abstract]) OR endocrine[Title/Abstract]) OR NET[Title/Abstract]	229180
#2	Add	Search digestive system neoplasms[MeSH Terms]	512405
#1	Add	Search neuroendocrine tumor[MeSH Terms]	145008

## Additional file

Additional file 1: Overview on primary outcomes and HRQoL results of studies included in systematic review. (DOCX 64 kb)

## Abbreviations

CONSORT: Consolidated standards of reporting trials
EORTC: European Organisation for research and treatment of cancer
FACIT: Functional assessment of chronic illness therapy
GEP-NET: Gastroenteropancreatic neuroendocrine tumours
HRQoL: Health-related quality of life
ISOQOL: International society for quality of life research
Norfolk QOL-NET: Norfolk quality of life – neuroendocrine tumor questionnaire
PRISMA: Preferred reporting items of systematic reviews and meta-analyses
PRO: Patient-reported outcome
QLG: Quality of life group
QLQ-C30: Quality of life questionnaire core 30
QLQ-GI.NET21: Quality of life questionnaire – neuroendocrine carcinoid
