# Supplementary material for: Systematic review reveals lack of quality in reporting health-related quality of life in patients with gastroenteropancreatic neuroendocrine tumours
Source: Health Qual Life Outcomes. 2016 Sep 10;14(1):127. doi: 10.1186/s12955-016-0527-2 (PMC5018190; doi:10.1186/s12955-016-0527-2)
Supplement: Additional file 1: — Overview on primary outcomes and HRQoL results of studies included in systematic review. (DOCX 64 kb) [file 12955_2016_527_MOESM1_ESM.docx]

Additional file 1. Overview on primary study outcomes and HRQoL results.

| Study | Primary outcome (intervention) | Brief summary of primary clinical outcomes | Brief summary of main HRQoL result |
| --- | --- | --- | --- |
| **Randomised controlled trials** | | | |
| Arnold et al., 2005 [[52](#_ENREF_52)] | PFS; long-term survival (octreotide vs. octreotide + INT) | - HR for PFS and long-term survival (4 year follow-up) in favour of combination therapy octreotide + INT | - overall QoL at 3-months assessment after start of therapy improved in octreotide group and decreased in combination therapy group - no results on other HRQoL domains reported |
| Bajetta et al., 2006 [[53](#_ENREF_53)] | non-inferiority (lan ATG vs. lan MP) | - no difference concerning symptomatic, biochemical, or objective responses | - similar mean scores for overall QoL^[[1]](#footnote-1)^ at baseline and 18 weeks (study end) for both groups - results on other HRQoL domains not interpretable due to wrong scoring |
| Caplin et al., 2014 [[42](#_ENREF_42)]  *(CLARINET)* | PFS (lan ATG vs. placebo) | - HR in favour of lan ATG | - no significant difference concerning overall QoL (24 months study period) - no results on other HRQoL domains reported |
| Jacobsen & Hanssen, 1995 [[54](#_ENREF_54)] | effect on clinical symptoms + tumour marker (octreotide vs. placebo) | - clinical outcome in favour of octreotide | - no significant difference concerning HRQoL (8 weeks study period) - significant improvement of social functioning and psychosocial distress following octreotide treatment (at 4 and 8 weeks) compared to baseline - no differences on other HRQoL domains at any assessment time point |
| Meyer et al., 2014 [[55](#_ENREF_55)] | OR (capecitabine + streptozocin vs. capecitabine + streptozocin + cisplatin) | - no significant difference in OR | - statistically and clinically significant reduction of overall QoL under capecitabine + streptozocin + cisplatin after 3 cycles (~9 weeks) compared to baseline - no significant reduction of overall QoL at 6-month follow-up in both groups compared to baseline (CAVE: drop-out rate) - no results on other HRQoL domains reported |
| Raymond et al., 2011 [[56](#_ENREF_56)] | PFS (sunitinib vs. placebo) | - PFS in favour of sunitinib | - statistically and clinically significant increase of diarrhoea and insomnia in sunitinib group (study period 10 cycles, i.e., ~10 months) - no differences on other HRQoL domains at any assessment time point |
| Rinke et al., 2009 [[57](#_ENREF_57)]  *(PROMID)* | TTP (octreotide LAR vs. placebo) | - TTP in favour of octreotide LAR | - no difference concerning overall QoL at baseline and at 6 months after baseline - no results on other HRQoL domains reported |
| Yao et al., 2016 [[45](#_ENREF_45)]  *(RADIANT-4)* | PFS (everolimus vs. placebo) | - PFS in favour of everolimus - AEs in 10% of patients (most common: stomatitis, diarrhoea, fatigue, infections, rash, peripheral oedema) | - no results on HRQoL reported |
| **Phase II studies** | | | |
| Bodei et al., 2011 [[58](#_ENREF_58)] | toxicity and efficacy (^177^Lu-DOTATATE) | - common acute events: mild asthenia - grade 1-2 haematological toxicity - response rate 29.4% - OS probability at 36 months: 68% | - improvement of fatigue, appetite loss, diarrhoea - results on other HRQoL domains not reported or not interpretable |
| Bushnell et al., 2010 [[59](#_ENREF_59)] | efficacy (^90^Y- DOTADOC) | - OR or stable disease in 74.4% | - durable improvements (minimum duration of 4 weeks) of usual activities in 24%, of anxiety/depression in 28%, in 29% of pain/discomfort, in 21% of mobility, and in 6% of self-care - significant linear improvement of health state (from baseline to estimate at 80 weeks) |

| Study | Primary outcome (intervention) | Brief summary of primary clinical outcomes | Brief summary of main HRQoL result |
| --- | --- | --- | --- |
| Claringbold et al., 2011 [[60](#_ENREF_60)] | safety and efficacy of capecitabine + ^177^Lu-octreotate | - 24% partial response, 70% stable disease, 6% progressive disease - >90% PFS at 2 years for patients with partial response and stable disease - symptomatic response in 42% of patients with diarrhea and flushing | - results on overall QoL not interpretable due to wrong scoring - no results on other HRQoL domains reported |
| Cwikla et al., 2010 [[61](#_ENREF_61)] | PFS, overall survival (^90^Y-DOTATATE) | - median PFS 17 months, median OS 20 months | - reduction of abdominal pain during or 4–8 weeks after therapy in 15/24 patients (recurrence of symptoms in 3 patients 10–19 months after end of therapy) - reduction of diarrhoea during or 4–8 weeks after therapy in 10/19 patients (normal bowel movements in 4 patients) - reduction of flushing after therapy in 9/11 patients; weight gain in 14/20 patients - no results on other HRQoL domains reported |
| Delpassand et al., 2014 [[62](#_ENREF_62)] | efficacy and safety (^177^Lu-DOTATATE) | - median PFS 16.1-16.5 months - radiological response in 31%, biochemical response in 32%, stable disease in 41%, progressive disease in 28% | - significant improvement of overall QoL from baseline across treatment to 3 months follow-up - no results on other HRQoL domains reported |
| Ducreux et al., 2014 [[43](#_ENREF_43)]  *(BETTER)* | PFS (5-FU/streptozocin + bevacizumab) | - median PFS 23.7 months - AEs of special interest in 85% of patients, most of these considered related to bevacizumab (proteinuria, bleeding/haemorrhage, hypertension) | - no significant changes in overall QoL from baseline to 3-, 6-, and 12-month follow-up - baseline median overall QoL score of 67 (on a scale of 0–100) remained mostly stable across entire study period |
| Mitry et al., 2014 [[44](#_ENREF_44)]  *(BETTER)* | PFS (capecitabine + bevacizumab) | - median PFS 23.4 months - targeted AEs in 37% of patients, most of these considered related to bevacizumab (mainly hypertension) | - no significant changes in overall QoL from baseline to 3-, 6-, and 12-month follow-up - median overall QoL score was 67 (on a scale of 0–100) at baseline, 63 at 6-month, and 71 at 12-month follow-up - at final visit 45% of patients showed no change in overall QoL |
| Frilling et al., 2006 [[63](#_ENREF_63)] | palliative benefits (^90^Y- and ^177^Lu-DOTATOC) | - stable disease in 11, partial response in 5, and progressive disease in 4 patients - no serious AE | - 10/16 patients with partial response or stable disease reported improvement in overall QoL |
| Khan et al., 2011 [[64](#_ENREF_64)] | HRQoL (^90^Y-DOTATATE) | N/A | - significant improvement of overall QoL, emotional and social functioning, insomnia, appetite loss, and diarrhoea after therapy - improvement of overall QoL and all symptoms of the QLQ-C30 in patients with remission (especially diarrhoea, pain, nausea/vomiting) - improvement of overall QoL and constipation in patients with stable disease |
| Korse et al., 2009 [[65](#_ENREF_65)] | investigation of CgA as biomarker for carcinoid syndrome | N/A | - significant correlations of CgA and physical functioning and overall QoL |
| Kulke et al., 2008 [[66](#_ENREF_66)] | radiologic response (sunitinib) | - overall OR rate: 16.7% in PNET and 2.4% in carcinoid patients | - no significant changes in health state during the first 6 treatment cycles - stable fatigue scores at each treatment cycle with transient, modest increases during dosing periods |
| Kvols et al., 2012 [[67](#_ENREF_67)] | efficacy (pasireotide) | - complete or partial symptom control (diarrhoea and flushing) in 27% | - descriptive statistics for physical well-being indicate stable scores in study completers - no analyses on other HRQoL domains reported |
| Martin-Richard et al., 2013 [[68](#_ENREF_68)] | PFS (lan ATG) | - median PFS was 12.9 months | - stable scores in all HRQoL domains (physical, emotional, social, role, and cognitive functioning, overall QoL, all symptoms) from baseline to week 56 |
| Study | Primary outcome (intervention) | Brief summary of primary clinical outcomes | Brief summary of main HRQoL result |
| Ruszniewski et al., 2004 [[69](#_ENREF_69)] | efficacy, safety (lan PR) | - improvement observed for 81% of flushing-target and 75% of diarrhoea-target patients at 6 months after baseline - AEs in 37% of patients | - significant decrease of self-reported diarrhoea from baseline to 2- and 6-month follow-up - no significant changes on other HRQoL domains |
| Wymenga et al., 1999 [[70](#_ENREF_70)] | efficacy, safety (lan PR) | - significant and permanent reduction of diarrhoea and flushing at month 1 and 6 after baseline | - baseline scores of all HRQoL domains were below population norms - significant improvement of emotional and cognitive functioning, overall QoL, fatigue, insomnia, and diarrhoea after 1 month of treatment - significant improvement of diarrhoea after 6 months of treatment compared to baseline - no significant change of other HRQoL domains after 6 months of treatment compared to baseline |
| Zuetenhorst et al., 2004 [[71](#_ENREF_71)] | clinical response (INT followed by unlabelled MIBG followed by ^131^I-MIBG) | - in 65% of patients stable disease after treatment with INT followed by unlabelled MIBG followed by ^131^I-MIBG (duration 34 weeks) | - no significant changes in functioning domains (physical, emotional, social, role, and cognitive functioning) at week 8 with INT monotherapy - significant reduction of flushes and worries concerning disease progression at week 8 with INT monotherapy - no significant changes in any of the HRQoL domains at week 21 after unlabelled MIBG - no HRQoL reported for weeks 21–34 with ^131^I-MIBG treatment |
| **Prospective studies** | | | |
| Fröjd et al., 2007 [[72](#_ENREF_72)] | HRQoL, psychosocial function | N/A | - significant and clinically meaningful difference between patients and Swedish population norms for role and social functioning, overall QoL, fatigue, and diarrhoea to the detriment of patients across entire study period (12 months) - highest symptom severity reported for fatigue, pain, dyspnoea, and diarrhoea, across entire study period - low levels of anxiety and depression across entire study period - significant and clinically meaningful linear improvement of role functioning - significant improvement of emotional functioning and significant increase of dyspnoea at months 7 and 12 |
| Fröjd et al., 2009 [[73](#_ENREF_73)] | satisfaction with care, HRQoL, anxiety, depression | N/A | - significant positive correlation of satisfaction with doctors’ care and HRQoL on all domains, including symptoms - higher satisfaction with doctors’ care (e.g., interpersonal skills, information provision) was related to improvements in all HRQoL domains, including symptoms, as well as in anxiety and depression |
| Haugland et al., 2013 [[74](#_ENREF_74)] | stress, self-efficacy, HRQoL (self-management intervention) | N/A | - significant improvement of stress, self-efficacy and physical health after self-management intervention (26 weeks duration) - no significant change of mental health score over time |
| Kalinowski et al., 2009 [[75](#_ENREF_75)] | safety, efficacy (^90^Yttrium microspheres) | - survival rates: 100% for 1 year, 57% for 2 and 3 years - 3 months after therapy partial response in 6/9 patients and stable disease in 3/9 patients - estimated TTP was 11.1 months | - results on HRQoL not interpretable due to wrong scoring |
| Kwekkeboom et al., 2003 [[76](#_ENREF_76)] | response, safety | - complete remission in 3%, partial remission in 35%, stable disease in 41%, progressive disease in 21% at 3-month follow-up (9 months since baseline) | - no significant change on functioning and symptom scales between baseline and 3-month follow-up - increasing percentage of patients who rated overall QoL >70 (on a scale of 0–100) between baseline and 3-month follow-up |
| Larsson & Janson, 2008 [[77](#_ENREF_77)] | HRQoL (erythropoietin) | N/A | - no statistically significant improvements in any of the HRQoL domains at months 4 and 8 or 24 compared to baseline |
| Study | Primary outcome (intervention) | Brief summary of primary clinical outcomes | Brief summary of main HRQoL result |
| Larsson et al., 2001 [[78](#_ENREF_78)] | HRQoL, anxiety, depression | N/A | - more impairment in role and emotional functioning, fatigue, nausea/vomiting, appetite loss, and diarrhoea at baseline compared to population norms - significant deterioration of physical functioning at 6, 9, and 12 months compared to baseline - significant reduction of nausea/vomiting and anxiety at 12 months compared to baseline - more impairment in physical and social functioning and overall QoL, fatigue, appetite loss, and diarrhoea at 12 months compared to population norms |
| O'Toole et al., 2000 [[79](#_ENREF_79)] | efficacy (octreotide vs. lanreotide) | - disappearance or improvement in the intensity of flushes in 68% of patients receiving octreotide and in 54% of patients receiving lanreotide - disappearance or improvement in diarrhoea in 50% of patients receiving octreotide and in 45% of patients receiving lanreotide | - no significant difference between patients who received octreotide and patients who received lanreotide on any of the HRQoL domains |
| Pasieka et al., 2004 [[80](#_ENREF_80)] | palliative benefits (^131^I-MIBG and ^111^In-octreotide) | - ^131^I-MIBG: symptomatic improvement in 12 and biochemical response in 2 patients; stability of tumour size in 6, regression in 2, and progression in 5 - ^111^In-octreotide: symptomatic benefit in 6 and biochemical response in 3 patients; stability of tumour size in 5, regression in 1, and progression in 5 | - ^131^I-MIBG group: 7/12 patients showed an improvement in HRQoL (assessed with an ad hoc questionnaire) when comparing pre-treatment to last follow-up score - ^111^In-octreotide group: 5/6 patients who reported a subjective improvement in their symptoms had an improvement in HRQoL |
| Spolverato et al., 2015 [[81](#_ENREF_81)] | HRQoL | N/A | - significant decrease in patients reporting diarrhoea (41.1% vs. 25.9%), flushing (34.1% vs. 10.5%), fatigue (36.5% vs. 28.2%), pain (20.0% vs. 10.6%), and skin discoloration (5.9% vs. 1.2%) from ‘before any treatment’ to ‘currently’ - significant decrease in patients reporting to be sad about being ill (31.8% vs. 23.2%) from ‘before any treatment’ to ‘currently’ - patients with a very poor HRQoL at the time of diagnosis were more likely to experience an improvement in HRQoL after treatment for neuroendocrine liver metastasis - many patients reported ongoing financial difficulties due to treatment |
| Teunissen et al., 2004 [[82](#_ENREF_82)] | HRQoL | N/A | - high functioning at baseline - highest impairment in fatigue, insomnia, pain, diarrhoea, and dyspnoea at baseline - significant increases in overall QoL, emotional, role, and social functioning at 6-week follow-up - significant decreases in fatigue, pain, insomnia, and diarrhoea at 6-week follow-up |
| **Cross-sectional studies** | | | |
| Beaumont et al., 2012 [[83](#_ENREF_83)] | HRQoL | - significantly more impairment in physical and social functioning, role limitation-physical, role limitation-emotional, general health, mental health, bodily pain, and vitality compared to US general population norms - lowest HRQoL in patients with both current NET and carcinoid syndrome, and in patients who experienced ≥4 bowel movements per day or ≥1 flushing episodes in a 2-week episode | |
| Pearman et al., 2016 [[84](#_ENREF_84)] | HRQoL | - patients with ≥4 bowel movements per day reported increasingly worse HRQoL - patients with 1 flushing episode per 14 days reported worse HRQoL than patients with no flushing; 2–3 flushing episodes per 14 days results in even worse mental and physical health - significant differences between treatment groups regarding global mental health, global physical health, fatigue, pain impact, physical function, and social role in favour of the surgery only group (vs. surgery + somatostatin vs. other treatments) - patients with recurrent disease had worse HRQoL compared to individuals with no current disease | |
| Gelhorn et al., 2016 [[85](#_ENREF_85)] | HRQoL, patient-reported symptom experiences | - more impairment in overall QoL, physical, role, and emotional functioning compared to US general population norms - most impairment in diarrhoea, fatigue, and insomnia - least impairment in constipation and nausea - unwillingness or inability to travel or participate in hobbies/usual activities as the most common concern related to symptoms of carcinoid syndrome | |
| Haugland et al., 2009 [[86](#_ENREF_86)] | HRQoL | - significantly more impairment in physical and social functioning, role limitation-physical, role limitation-emotional, general health, mental health, and vitality compared to Norwegian general population norms | |
| Study | Primary outcome (intervention) | Brief summary of main HRQoL result | |
| Haugland et al., 2016 [[87](#_ENREF_87)] | self-efficacy, social support, cancer-related stress, physical HRQoL | - age directly and negatively related correlated with physical HRQoL, self-efficacy, and social support - older patients with less optimistic self-beliefs experienced lower levels of physical HRQoL - cancer-related stress not significantly associated with physical or mental HRQoL | |
| Larsson et al., 1998 [[88](#_ENREF_88)] | HRQoL | - high functioning scores - most impairment in fatigue, dyspnoea, and diarrhoea | |
| Larsson, Sjöden et al., 1999 [[89](#_ENREF_89)] | HRQoL | - high functioning scores - most impairment in fatigue, diarrhoea, and dyspnoea - patients with carcinoids experienced significantly more diarrhoea, less pain and sleeping problems than patients with endocrine pancreatic tumours - patients under ongoing treatment reported more problems with diarrhoea than did those without current treatment | |
| Larsson, von Essen et al., 1999 [[90](#_ENREF_90)] | HRQoL, anxiety, depression | - high functioning scores - most impairment in fatigue, dyspnoea, diarrhoea - least impairment in nausea/vomiting and constipation - patients with carcinoids reported more problems with diarrhoea than did patients with endocrine pancreatic tumours - overall, levels of anxiety and depression were low; patients reporting clinically relevant levels of anxiety and depression also reported lower functioning and a higher level of symptoms | |
| Larsson et al., 2003 [[91](#_ENREF_91)] | psychosocial function, HRQoL | - interview data revealed several HRQoL aspects not included in the QLQ-C30: disease-specific symptoms and side-effects, worries about future, good appearance, and live one’s life in accordance with one’s desire | |
| Larsson et al., 2007 [[92](#_ENREF_92)] | HRQoL | - high functioning scores - most impairment in fatigue | |
| Petzel et al., 2012 [[93](#_ENREF_93)] | fear of cancer recurrence, HRQoL, psychosocial distress | - clinically important level of fear of cancer recurrence was associated with low HRQoL in all domains as well as with high anxiety | |
| Pezzilli et al., 2009 [[94](#_ENREF_94)] | HRQoL, anxiety, depression | - no significant difference regarding physical health aspects between patients and the general population - significantly lower mental health in patients compared to normative data | |
| Pezzilli et al., 2010 [[95](#_ENREF_95)] | HRQoL | - physical and mental health aspects indicated a relatively good HRQoL and did not differ significantly from those of the Italian general population | |
| Ruszniewski et al., 2016 [[96](#_ENREF_96)]  *(SYMNET)* | patient-reported satisfaction with diarrhoea control | - 76% of patients were ‘completely satisfied’ or ‘rather satisfied’ with diarrhoea control at study visit (= routine clinic visit) - 79% of patients reported improved diarrhoea after treatment with lan ATG/Depot - high functioning scores and overall QoL - most impairment in fatigue, insomnia, diarrhoea, muscle/bone pain, and disease-related worries - least impairment in vomiting, dyspnoea, appetite loss, constipation, and financial difficulties - more patients who were satisfied with diarrhoea control also reported good to excellent overall QoL (70%) compared with patients who were dissatisfied (39%) or neither satisfied nor dissatisfied (48%) | |
| van der Horst-Schrivers et al., 2009 [[97](#_ENREF_97)] | sexual function, HRQoL | - only descriptive statistics for HRQoL data - 8/27 of the male patients reported a clinically relevant sexual problem (arousal, erection or orgasm); only 1/16 woman reported sexual dysfunction - prevalence of sexual dysfunction similar to a Dutch reference population | |
| von Essen et al., 2002 [[98](#_ENREF_98)] | satisfaction with care, HRQoL, anxiety, depression | - satisfaction with psychosocial care was associated with better overall QoL and psychosocial function - patients experiencing a clinically important level of anxiety were less satisfied with several care aspects (e.g., nurses’ communication skills, doctors’ interpersonal skills) than those experiencing less anxiety | |
| Note. adverse events (AEs); Chromogranin A (CgA); hazard ratio (HR); health-related quality of life (HRQoL); interferon-α (INT); lanreotide autogel (lan ATG); lanreotide microparticles (lan MP); lanreotide prolonged-release (lan PR); meta-iodbenzylguanidin (MIBG); neuroendocrine tumours (NET); objective response (OR); octreotide long-acting repeatable (octreotide LAR); overall survival (OS); pancreatic NET (PNET); patient-reported outcomes (PROs); partial response (PR); progression-free survival (PFS); progressive disease (PD); quality of life (QoL); Quality of Life Questionnaire Core30 (QLQ-C30); stable disease (SD); time to progression (TTP). | | | |

1. questionnaire scale assessing global health status/QoL [↑](#footnote-ref-1)
